# A Simplified SSY Estimate Method to Determine EPFM Constraint Parameter for Sensor Design †

**DOI:** 10.3390/s19030717

**Published:** 2019-02-10

**Authors:** Ping Ding, Xin Wang

**Affiliations:** 1 National Research Base of Intelligent Manufacturing Service, Chongqing Technology and Business University, Chongqing 400067, China; 2 Department of Mechanical and Aerospace Engineering, Carleton University, Ottawa, ON K1S 5B6, Canada

**Keywords:** sensor design, sensor structure analysis, two parameter approach, constraint parameter, small scale yielding, *T*-stress, estimate method, simplified format, biaxial loading

## Abstract

To implement a sensor structure analysis and design (as well as other engineering applications), a two-parameter approach using elastic–plastic fracture mechanics (EPFM) could be applied to analyze a structure more accurately than a one-parameter approach, especially for structures with low crack constraint. The application of the *J-A* two-parameter approach on sensors and other structures depends on the obtainment of a constraint parameter *A*. To conveniently and effectively obtain the *A* parameter values, the authors have developed a *T*-stress-based estimate method under a small-scale yielding (SSY) condition. Under a uniaxial external loading condition, a simplified format of the *T*-stress-based estimate has been proposed by the authors to obtain the parameter *A* much more conveniently and effectively. Generally, sensors and other practical engineering structures endure biaxial external loading instead of the uniaxial one. In the current work, the simplified formation of the estimate method is extended to a biaxial loading condition. By comparing the estimated *A* parameter values with their numerical solutions from a finite element analysis (FEA) results, the extension of the simplified formation of *T*-stress-based estimate method to biaxial loading was discussed and validated. The comparison procedure was completed using a wide variety of materials and geometrical properties on three types of specimens: single edge cracked plate (SECP), center cracked plate (CCP), and double edge cracked plate (DECP).

## 1. Introduction

The service life and reliability of sensors greatly affect the reliability of a prognostics and health management (PHM) system [1,2,3], just like a battery management system. To ensure sensor service life and reliability, an elastic–plastic fracture mechanics (EPFM) analysis of sensor structures is a significant step in sensor structure analysis and design, especially for a sensor bracket structure.

Traditionally, a sensor structure EPFM analysis is based on a one-parameter *J*-based approach [4,5], where only the applied external load is described by a *J*-integral parameter [6]. However, this only works well for sensor structures under high constraint conditions. Under low constraint conditions, the constraint effect on near-tip stress and displacement fields cannot be ignored. Under these circumstances, a one-parameter approach usually overestimates the stress values of sensor structures. It leads to an inappropriate structure maintenance strategy, and results in unnecessary component replacements and labor costs accordingly.

To describe the constraint effect, a second parameter needs to be introduced to the EPFM approach. Thus, several two-parameter approaches have been developed based on the application the *J*-integral for loading description. By extending a Williams solution [7] from an elastic material to an elastic–plastic material, the *J-T* two-parameter approach was suggested by Betegon and Hancock [8] as well as Al-Ani and Hancock [9], with *T*-stress illustrating the constraint effect. Using term *Q* as the constraint (and second) fracture parameter, the *J-Q* two-parameter approach was proposed by O’Dowd and Shih [10,11]. Yang et al. [12] developed the *J-A*_2_ two-parameter approach, whose three-term expansion includes a *J*-integral and a second fracture parameter *A*_2_. As an alternative format to the *J-A*_2_ approach, a *J-A* two-parameter approach was derived by Nikishkov et al. [13,14], in which the second parameter *A_2_* is replaced by *A*. In a wide variety of structure geometry configurations and external loading conditions, all two-parameter approaches (*J-T*, *J-Q* and *J-A*_2_ (*A*)) provide an effective characterization of elastic–plastic crack-tip (-front) fields [9,11,12,14].

To apply the EPFM two-parameter approaches to both the sensor structure analysis and design, the values of the two parameters, *J*-integral and the constraint parameter (*T*-stress, *Q*, or *A*_2_ (*A*)) for the considered sensor components, must first be obtained. Solutions of the *J*-integral had been well established in the early developing stage of EPFM. Research efforts now focus on the obtainment of constraint parameter solutions since the development of two-parameter approaches. As a linear elastic constraint parameter, both numerical and analytical solutions of *T*-stress have been well developed [15,16]. Dealing with material nonlinearities, the solutions of elastic–plastic constraint parameters (*Q* and *A*_2_ (*A*), etc.) have not been well established, which depends on external loading conditions, material hardening characteristics, and structure geometry. 

Through their original definitions, theoretically, the solutions of *Q* and *A*_2_ (*A*) parameters could be determined numerically, based on results of finite element analysis (FEA). A “point match” method was used by Yang et al. [12] to numerically determine the *A_2_* parameter values. A fitting method is suggested by Nikishkov et al. [14] to determine the *A* solution from FEA results. However, determining parameter *A* values through numerical methods is not effective for any engineering application, as for example, for a fracture mechanics analysis of a sensor supporting structure. The reason for this is that for a wide range of material, geometrical, and loading properties, the numerical determination of *Q* and *A*_2_ (*A*) is a time-consuming endeavor. Systematically developing estimate (prediction) methods for values of constraint parameters *A*_2_ (*A*) and *Q* are necessary to apply EPFM two-parameter approaches conveniently and effectively to practical sensor engineering problems and theoretical investigation.

Both numerical and analytical solutions to elastic *T*-stress have been well established, as mentioned in previous paragraphs. It is possible to obtain constraint *A* parameter values directly from *T*-stress, as long as the relationship between parameter *A* and *T*-stress is determined. As mentioned, the estimate method for a constraint parameter should be valid under small-scale yielding (SSY) cases, as *T*-stress is a linear elastic parameter. 

An estimate method for constraint parameter *A* under SSY has already been developed by authors [17] to predict *A* values from *T*-stress conveniently and quickly. Its simplified formation has also been obtained by authors [18] based on the shape similarity of *A-T* curves. The proposed *T*-stress-based estimate method for parameter *A* and its simplified format are both developed based on uniaxial external loading conditions. The load applied on a real component, specifically, a sensor supporting bracket, is generally biaxial.

In the current work, the simplified format of the *T*-stress-based estimate method is extended to a biaxial loading condition through the specimen analyses of single edge cracked plates (SECP), center cracked plates (CCP) and double edge cracked plates (DECP). Theoretical backgrounds will be illustrated in Section 2. The FEA process and numerical solutions of constraint parameter *A* will be discussed under SSY in Section 3. In Section 4, the simplified format of the *T*-stress-based estimate method will be extended to a biaxial loading condition with biaxial ratios *λ* = 0.5, 1.0, and the estimated solutions of constraint parameter *A* will be compared with the corresponding FEA numerical solutions. In Section 5, concluding remarks will be given. 

## 2. Theoretical Background

### 2.1. J-T and J-A Two-Parameter Approach

For two-dimensional crack-tip stress fields, Williams [7] suggested a series solution,

(1) $$\sigma_{ij}(r,\theta )=\frac{K_{}}{\sqrt{2\pi .r}}f_{ij}(\theta )+T\delta_{1i}\delta_{1j}$$

where *T* is a uniform stress parallel to the crack face, namely *T*-stress. *K* is the stress intensity factor. (*r, θ*) are polar coordinates, with the origin located at the crack tip. *f_ij_* (*θ*) are the non-dimensional angular functions; *δ*_1*i*_ and *δ*_1*j*_ are Kronecker deltas, with a range of 1–2 for indices *i* and *j*. The expression is the so-called *K-T* two-parameter approach for a linear elastic material.

Betegon and Hancock [8] as well as Al-Ani and Hancock [9] extended the *K-T* approach from an elastic material to an elastic–plastic material range and suggested a EPFM *J-T* two-parameter approach, where, by keeping the *T*-stress for constraint effect description, *K* is replaced by a *J*-integral to characterize the loading level in elastic-plastic material. The *J-T* approach is generally only suitable for an SSY condition with *T*-stress being a parameter of linear elastic fracture mechanics 

Another important EPFM approach is the *J-A*_2_ (or *J-A*) two-parameter approach with a three-term asymptotic expansion controlled by two fracture parameters. *J* and *A*_2_, the *J-A*_2_ approach is first proposed by Yang et al. [12]. An alternative format of the *J-A*_2_ approach, the *J-A* approach, is derived by Nikishkov et al. [13,14], where the constraint parameter *A*_2_ in *J-A*_2_ approach is replaced by its alternative normalized form, *A*. The *J-A* approach is the one utilized by the present authors.

When hardening exponent *n ≥* 3 according to the formula expressions and variable terminologies used by Nikishkov et al. [13,14], a *J-A* three-term asymptotic solution for crack-tip stress fields could be written as:

(2) $$\frac{\sigma_{ij}}{\sigma_{0}}=A_{0}{\bar{r}}^{s}{\bar{\sigma }}_{ij}^{\left(0\right)}(\theta )-A{\bar{r}}^{t}{\bar{\sigma }}_{ij}^{\left(1\right)}(\theta )+\frac{A^{2}}{A_{0}}{\bar{r}}^{2t-s}{\bar{\sigma }}_{ij}^{\left(2\right)}(\theta )$$

where *σ*_0_ is yield stress; *σ_ij_* (*θ*) are stress components *σ_r_*, *σ_θ_* or *σ_rθ_* in the polar coordinate system with origin at the crack tip; and ${\bar{\sigma }}_{ij}^{\left(0\right)}(\theta )$, ${\bar{\sigma }}_{ij}^{\left(1\right)}(\theta )$ and ${\bar{\sigma }}_{ij}^{\left(2\right)}(\theta )$ are normalized angular functions. The amplitude *A*_0_ is expressed as *A*_0_ = (α*ε*_0_*I_n_*)^−1/(*n*+1)^, which is determined based on a mathematical derivation [13]. Here, *ε*_0_ is yield strain, α is material coefficient, and *I_n_* is a scaling integral only depending on material hardening exponent *n* (see [5,6] for details). The dimensionless radius $\bar{r}$ is defined as $\bar{r}=r/\left(J/\sigma_{0}\right)$, where *J* is the *J*-integral at the crack tip. The power *t* is an eigenvalue depending on hardening exponent *n*, while the power *s* is defined as *s* = −1/(*n* + 1). The values of asymptotic power *t*, scaling integral *I_n_* and normalized angular functions ${\bar{\sigma }}_{ij}^{\left(0\right)}(\theta )$, ${\bar{\sigma }}_{ij}^{\left(1\right)}(\theta )$ and ${\bar{\sigma }}_{ij}^{\left(2\right)}(\theta )$ could be determined through a computational algorithm developed by Nikishkov [13]. 

Equation (2) indicates that a three-term expansion could be used to describe the crack tip field, which is controlled by two parameters, the magnitude of the first term (*J*-integral) and a second parameter (*A*) in the second and third terms. Through determining the values of the two parameters *J* and *A*, crack tip stress components of structures could be easily obtained from Equation (2).

To determine crack-tip stress fields of sensor structures through Equation (2), the solutions of the *J*-integral and second parameter *A* must first be obtained. The ways to determine *J*-integral values have been well established. Unfortunately, methods for the value obtainment of constraint parameter *A* are still quite scarce. In next section, an estimate method developed by the authors [17] will be presented, which predicts constraint parameter *A* values from *T*-stress both conveniently and effectively. 

### 2.2. T-Stress-Based Estimate of Constraint Parameter A

We [17] analytically proved the existence of a one-to-one relationship between constraint parameter *A* and *T*-stress under an SSY (low load) condition,

(3) $$A=A_{T}\left(\frac{T}{\sigma_{0}},n\right)$$

where the symbol *A_T_* indicates the constraint parameter *A* solutions obtained from the *T*-stress directly.

A detailed expression of the one-to-one *A-T* relationship (Equation (3)) can be obtained by the least square fitting method based on the parameter *A* solutions, which are obtained from FEA results of modified boundary layer (MBL) formulation for various *T*-stress values (see Section 3 for details). One could express the detailed *A-T* relationship as follows [17],

(4) $$A_{T}\left(\frac{T}{\sigma_{0}},n\right)=A_{SSY}(n)+m_{1}(n)\left(\frac{T}{\sigma_{0}}\right)+m_{2}(n){\left(\frac{T}{\sigma_{0}}\right)}^{2}+m_{3}(n){\left(\frac{T}{\sigma_{0}}\right)}^{3}.$$

A third-order polynomial could be used to represent the detailed one-to-one *A-T* relationship under the SSY condition for each case of a hardening exponent *n* value, see Equation (4). The values of polynomial coefficients *m*_1_(*n*), *m*_2_(*n*), and *m*_3_(*n*) are determined through a fitting process, which depend on the *n* values. *A_SSY_*(*n*) denotes the constraint parameter *A* value under the small-scale yielding (SSY) condition when the *T*-stress value equals zero (*T* = 0). 

In Equation (4), *T*-stress is normalized by yield stress *σ*_0_, *T*/*σ*_0_. It could be rewritten as follows for the cases that sensor structures with the external load ratio *σ*/*σ*_0_ [17]:

(5) $$\frac{T}{\sigma_{0}}=\frac{T}{\sigma }\cdot \frac{\sigma }{\sigma_{0}}=V\left(\frac{a}{W}\right)\cdot \frac{\sigma }{\sigma_{0}}$$

where *σ*_0_ is the yield stress, *V = T*/*σ*, *a* is specimen crack length and *W* specimen width, and *V* is the normalized *T*-stress by external load *σ*.

A detailed expression of the *A-T* relationship could be obtained for sensor crack structures with external loading ratio *σ*/*σ*_0_, by combining Equation (5) with (4) [17]:

(6) $$A_{T}\left(\frac{\sigma }{\sigma_{0}},\frac{a}{W},n\right)=A_{SSY}(n)+\left(\frac{\sigma }{\sigma_{0}}\right)g_{1}(\frac{a}{W},n)+{\left(\frac{\sigma }{\sigma_{0}}\right)}^{2}g_{2}(\frac{a}{W},n)+{\left(\frac{\sigma }{\sigma_{0}}\right)}^{3}g_{3}(\frac{a}{W},n)$$

where *g_i_* (*a/W*, *n*) = [*V*(*a/W*)]*^i^ m_i_* (*n*), with *i* = 1, 2, 3 for term *g_1_*, *g_2_*, and *g_3_*, respectively. *A_SSY_*(*n*), which is the parameter *A* value under SSY condition for *T* = 0 case, is used here to closely approximate the *A* value when external load ratio (*σ*/*σ*_0_) is very small. By determining values of polynomial coefficients *m*_1_(*n*), *m*_2_(*n*), and *m*_3_(*n*), and the normalized *T*-stress, *V*, constraint parameter *A* under SSY can be conveniently obtained through Equation (6) for various models including specimens SECP, CCP, and DECP, as well as for final practical sensor structures.

## 3. Finite Element Analysis and Numerical Solution of Constraint Parameter *A*

### 3.1. Modified Boundary Layer Problem

**Figure 1:**
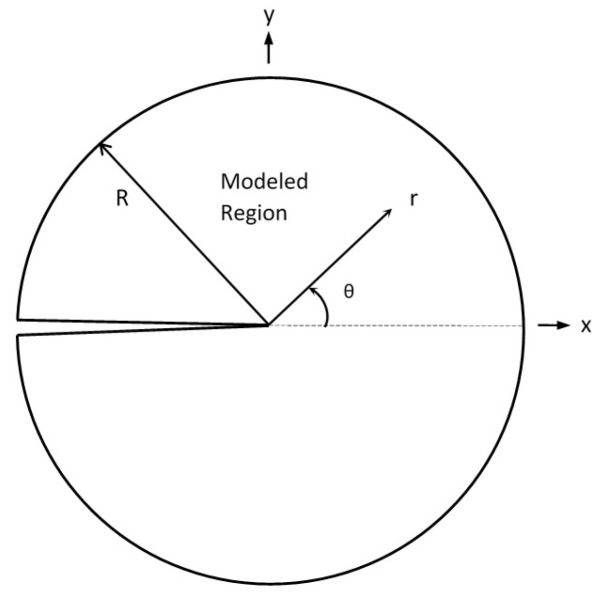
The modified boundary layer problem model.

To characterize the small-scale yielding (SSY) condition of cracked models (Figure 1), the modified boundary layer (MBL) formulation is usually used. By combining *J-T* and *K-T* two-parameter approaches, the MBL simulation is a practical investigating application. 

A typical MBL model is given in Figure 1, where *R* denotes the model maximum radius. As an elastic–plastic near crack-tip (-front) problem, the MBL formulation has elastic boundary conditions; that is, far-field stress intensity factor *K* and far-field *T*-stress characterize its asymptotic boundary stress field. Through the stress intensity factor *K* and *T*-stress, loadings applied on an MBL model are represented using displacement boundary conditions. At the far-field boundary of model, *r_max_* = *R*, the loadings are applied uniformly on the MBL finite element model. The values of plane strain displacement components *u_x_* and *u_y_* could be calculated based on the *K-T* stress fields formula [17,19],

(7a) $$u_{x}=\frac{K}{2\mu }\sqrt{\frac{r}{2\pi }}\cos\left(\frac{\theta }{2}\right)\left[\kappa -1+2\sin^{2}\left(\frac{\theta }{2}\right)\right]+\frac{1-\nu }{2\mu }Tr\cos\theta$$

(7b) $$u_{y}=\frac{K}{2\mu }\sqrt{\frac{r}{2\pi }}\sin\left(\frac{\theta }{2}\right)\left[\kappa +1-2\cos^{2}\left(\frac{\theta }{2}\right)\right]+\frac{\left(-\nu \right)}{2\mu }Tr\sin\theta$$

where *ν* is Poisson’s ratio, parameter *κ* = 3 − 4*ν*, and *μ* is the shear modulus. The far-field stress intensity factor *K* for plane strain could be obtained based on the far-field *J*-integral value through the relationships between them,

(8) $$K=\sqrt{\frac{JE}{1-\nu^{2}}}$$

where *E* is the Young’s modulus.

### 3.2. Parameter A Numerical Solutions under SSY

The deformation theory of plasticity is used as a material model for the FEA of the MBL problem as well as for the SECP, CCP, and DECP specimen. The Ramberg–Osgood power-law strain hardening relation is included in the commercial finite element code ABAQUS [20]. The Ramberg–Osgood relation for uniaxial stress-strain curve could be represented as,

(9) $$\frac{\varepsilon }{\varepsilon_{0}}=\frac{\sigma }{\sigma_{0}}+\alpha {\left(\frac{\sigma }{\sigma_{0}}\right)}^{n}$$

where the relation between material yield strain ε_0_ and the yield stress *σ*_0_ is *ε*_0_ = *σ*_0_/*E*, *α* is a material coefficient, and the material hardening exponent *n* should be greater than 1. 

The finite element code ABAQUS was utilized to implement all finite element analyses. For the finite element analyses of MBL and SECP, CCP, DECP, the material properties are: elasticity modulus *E* = 2.0 × 10^11^ Pa; Poisson ratio *ν* = 0.3; yield stress *σ*_0_ = 4.0 × 10^8^ Pa; material coefficient *α* = 1.0, and hardening exponent *n* = 3, 4, 5, 7, 10. A wide range of strain hardening behaviors were covered.

To simulate the SSY (low load) condition of MBL problem, the far-field *J*-integral value was fixed as 1.0 × 10^4^ J/m^2^. It meanwhile determined the value of far-field stress intensity factor *K* through Equation (8). With various hardening exponents *n* = 3, 4, 5, 7, 10, finite element analyses of MBL formulation were implemented for different *T*-stress values, *T*/*σ*_0_ = −0.8, −0.6, −0.4, −0.2, 0.0, 0.2, 0.4, 0.6, 0.8, respectively.

**Figure 2:**
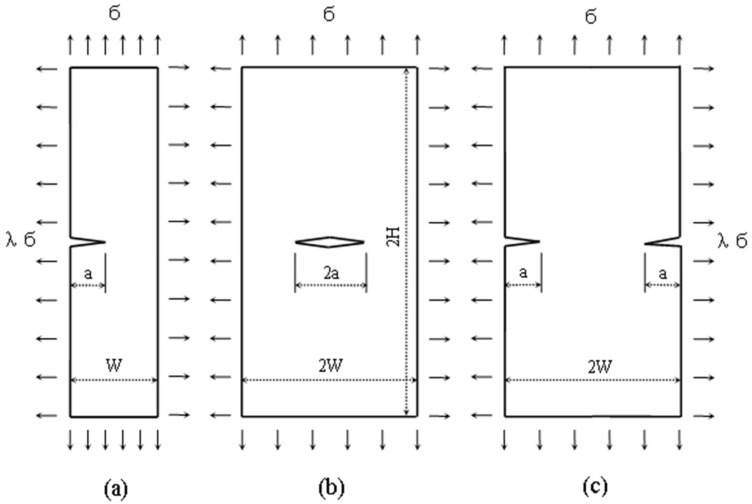
Plane-strain crack specimens under biaxial loading, (**a**) SECP, (**b**) CCP, (**c**) DECP.

For the finite element analyses of the three specimens at hand (SECP, CCP, and DECP), some geometrical properties were applied. *H/W* is the ratio of specimen length *H* to width *W*, which is fixed as 1.875 here. Specimen finite element models were investigated based on various ratios of crack length to specimen width *a*/*W* = 0.1, 0.3, 0.5, 0.7. See Figure 2 for reference. Biaxial loading were applied on the four edges of the three specimen models, with biaxial ratios *λ* = 0.5, 1.0.

For all cases of MBL and SECP, CCP, and DECP, the *J*-integral values were determined through domain integral method [21], which is included in the commercial code ABAQUS [20]. Based on FEA results, constraint parameter *A* values could be numerically determined using a fitting method suggested by Nikishkov et al. [14]. See reference [14] and [17,19] for more details about the procedure of the fitting method. 

**Figure 3:**
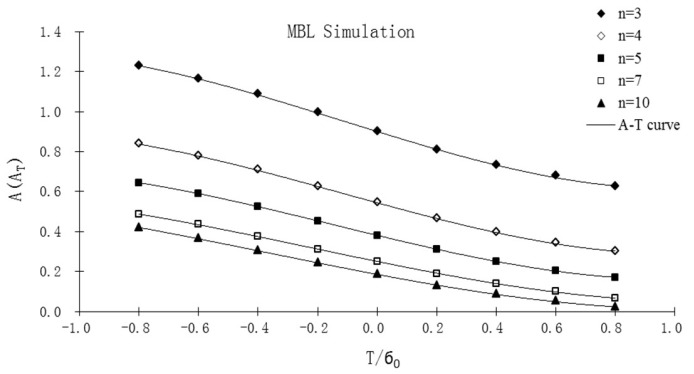
Curves of *A* values from FEA results vs. normalized *T*-stress, *T*/*σ*_0_, for MBL formulation.

Table 1 illustrates the numerical solutions of constraint parameter *A* for MBL simulation under SSY. By fitting the *A* numerical solutions for MBL (Table 1), the *A-T* relationship curves for various *n* values could be obtained, which are shown in Figure 3. The *A-T* curves could be used to determine the coefficients *m_i_*(*n*) of Equation (4), see next section. For the three crack specimens (SECP, CCP, and DECP), the constraint parameter *A* values determined from FEA results vary with biaxial ratios *λ* = 0.5, 1.0 and normalized external load, *σ*/*σ*_0_.

## 4. Simplified Format of *T*-Stress-Based Estimate Method under Biaxial Loading

### 4.1. Simplified Formation of T-Stress-Based Estimate

The *T*-stress-based estimate method suggested by the authors to obtain constraint parameter *A* values directly from *T*-stress was applied under SSY conditions (see [17]). In addition, its simplified format was successfully developed based on a uniaxial external loading condition [18]. In our current work, the simplified formation of the *T*-stress-based estimate was extended to sensor structures with a biaxial external loading condition.

A phenomenon was observed in the MBL FEA process in authors’ previous work [17]. Referring Figure 3, it can be found that, the shape of *A* vs. *T*/*σ*_0_ curves (MBL *A-T* curves) for various hardening exponent *n* values is similar. The *A-T* curves for different *n* values are “parallel”, and only differ from each other by constant parameter *A* values, *A_SSY_*(*n*). 

Therefore, once the *A* vs. *T*/*σ*_0_ curve of MBL formulation for any specified *n* is obtained, it could be used to predict the *A* vs. *T*/*σ*_0_ curves for other *n* values (see Figure 3). With the obtained parameter *A* values for *n* = 10, for example, *A* solutions of MBL formulation for any other *n* value could be obtained from the *T*-stress using following formula [18],

(10) $$A_{T}\left(\frac{T}{\sigma_{0}},n\right)=A_{SSY}\left(n\right)+\left\{A_{T}\left(\frac{T}{\sigma_{0}},n=10\right)-A_{SSY}\left(n=10\right)\right\}$$

Combining Equations (4) and (10), a simplified format of Equation (4) could be obtained with condition of hardening exponent *n* = 10,

(11) $$A_{T}\left(\frac{T}{\sigma_{0}},n\right)=A_{SSY}(n)+m_{1}(n=10)\left(\frac{T}{\sigma_{0}}\right)+m_{2}(n=10){\left(\frac{T}{\sigma_{0}}\right)}^{2}+m_{3}(n=10){\left(\frac{T}{\sigma_{0}}\right)}^{3}$$

Here, coefficients *m*_1_, *m*_2_ and *m*_3_ for the *n* = 10 case are denoted as *m*_1_ (*n* = 10), *m*_2_ (*n* = 10) and *m*_3_ (*n* = 10).

Meanwhile, combining Equation (5) with (11), Equation (6) could be rewritten for the specimen and sensor structure models with normalized external load σ/σ_0_ as variable,

(12) $$A_{T}\left(\frac{\sigma }{\sigma_{0}},\frac{a}{W},n\right)=A_{SSY}(n)+\left(\frac{\sigma }{\sigma_{0}}\right)g_{1}(\frac{a}{W},n=10)+{\left(\frac{\sigma }{\sigma_{0}}\right)}^{2}g_{2}(\frac{a}{W},n=10)+{\left(\frac{\sigma }{\sigma_{0}}\right)}^{3}g_{3}(\frac{a}{W},n=10)$$

where *g_i_* (*a/W*, *n*) = [*V*(*a/W*)]*^i^ m_i_* (*n* = 10), with *i* = 1, 2, 3.

### 4.2. Determining Constraint Parameter A under Biaxial Loading

Under SSY conditions, the simplified *T*-stress-based estimate method (Equation (12)) could be used to predict the parameter *A* directly from *T*-stress for the analysis of sensor structures and specimens—such as SECP, CCP, and DECP—based on the obtainment of *A_SSY_* (*n*) solutions, coefficients *m_i_* (*n*) values, and normalized *T*-stress, *V* (*V = T/σ*). 

As mentioned in Section 2.2, *A_SSY_* (*n*) solutions are the parameter *A* values of MBL for *T* = 0 cases, which are available in Table 1. Based on Equation (4), coefficients *m_i_* (*n*) values could be determined from parameter *A* numerical solutions of MBL (Table 1 or Figure 3) for various *n* values through the least square fitting process. The *A_SSY_* (*n*) solutions and coefficients *m_i_* (*n*) values for various *n* values [17,19] are listed in Table 2. 

In practical application, the *T*-stress values of structures vary with external load. To effectively apply the *T*-stress-based estimate method for parameter *A*, *T*-stress solutions need to be obtained conveniently and quickly through estimation instead of the time-consuming numerical computation (FEA). Through a *T*-stress superposition estimation method, the weight function method suggested by Wang [16], the *T*-stress and its normalized value *V* could be obtained. The proposed weight functions are highly accurate, which are originally developed through FEA.

(13) $$T={\int_{0}^{a}\sigma (x)w(x,a)dx+\sigma (\lambda -1)|}_{x=a}$$

Here, *σ*(*x*) is the stress distribution on the crack face and w(*x*, *a*) is the weight function for the *T*-stress; *σ* is the applied far field load (see Figure 2); λ is the biaxial loading ratio. 

The expression of stress distribution *σ*(*x*) depends on the applied external load, which is available in reference [16] for various external load types. For current uniform external load condition, the stress on crack face is equal to the external load, that is, *σ*(*x*) = *σ*. The weight function w(*x*, *a*) can be expressed as,

(14) w(x,a)=2πa[D1(1−xa)12+D2(1−xa)32]

The coefficients *D*_1_ and *D*_2_ in Equation (14) can be determined through,

(15a) $$D_{1}=\frac{15}{16}\pi (5V_{0}-7V_{1})$$

(15b) $$D_{2}=\frac{5}{16}\pi (35V_{1}-21V_{0})$$

The values of coefficients *V*_0_ and *V*_1_ depend on structure geometry, such as SECP, CCP, and DECP specimens, which could be found in reference [16]. With the obtained *T*-stress values, the normalized *T*-stress, *V*, can be determined through *V = T/σ*.

Through the weight function method (Equation (13)), the normalized *T*-stress values (*V*) for SECP, CCP, and DECP in current work were obtained. See reference [16] for more details about the weight function method and the *T*-stress determining procedure.

### 4.3. Validation and Discuss

Through the simplified format of the *T*-stress-based estimate method (Equation (12)) and corresponding coefficient values (Table 2), solutions of constraint parameter *A* for SECP, CCP, and DECP specimens were determined directly from the normalized *T*-stress, *V*. As the *T*-stress-based estimate method was only valid under an SSY condition, the maximum applicable external load (applicability range) of simplified format estimate (Equation (12)) should be discussed. With 10% as the acceptable prediction error, the maximum applicable load ratio (*σ*/*σ*_0_) of the simplified format estimate method under biaxial loading are listed in Table 3.

**Figure 4:**
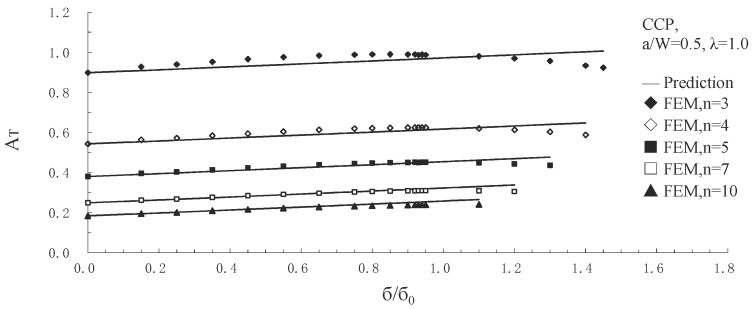
Comparisons of predicted *A* values from *T*-stress with FEA data for CCP, *a/W* = 0.5, *λ* = 1.0.

The predicted *A* values through a simplified format of the *T*-stress-based estimate method (Equation (12)) for biaxial ratio *λ* = 0.5 and 1.0 were compared with those solutions obtained from FEA results. Based on the relative crack length *a/W* = 0.1, 0.3, 0.5, 0.7 with various hardening exponents *n* = 3, 4, 5, 7, and 10, respectively, the comparisons for SECP, CCP, and DECP were implemented extensively. As an example, Figure 4 illustrates *A* value comparison for CCP with *a/W* = 0.5 and *λ* = 1.0.

When external load ratios are not greater than the maximum applicable ones (Table 3), for SECP, CCP, and DECP with *λ* = 0.5, 1.0, most differences between predicted *A* values and its FEA solutions are less than 5%. The maximum difference overall is 10.99% for all three specimens with the two biaxial ratios, showing good agreement. 

The maximum applicable external load (applicability range) of the original format of *T*-stress-based estimate method (Equation (6)) are listed in Table 4, which has been obtained in previous work [19]. Comparing the current maximum applicable loads of simplified format estimate method (Table 3) with those from original format estimate (Table 4), it could be found that, for the SECP, CCP, and DECP specimens, generally, the applicability range (maximum applicable loads) of the simplified format (Equation (12)) is slightly smaller than that of the original format (Equation (6)). Current simplified format (Equation (12)) is based on *n* = 10, thus a decrease of the applicability range only occurs for *n* = 3, 4, 5 and 7. The same phenomenon was found for the cases under a uniaxial loading condition (see [18]). 

The reason for the reduced applicability range is the same as that of the simplified format *T*-stress-based estimate applied under uniaxial loading condition [18]: the curves of the *A* value vs. normalized *T*-stress, *T*/*σ*_0_, in Figure 3 are not identical in shape, they are only “similar”. However, only one curve shape for a specified hardening exponent *n*, for example *n* = 10, was utilized in the simplified format estimate method (Equations (11) and (12)) to predict *A* solutions for other *n* values.

Just like for a uniaxial loading condition [18], a biaxial loading, although with the reduced applicability range, can use the simplified formation of *T*-stress-based estimate method to estimate *A* solutions for other *n* values based on available FEA results of MBL formulation for arbitrary single value of *n*. The simplified format of the *T*-stress-based estimate method enables determining *A* values from *T*-stress much more conveniently and quickly than its original formation for sensor components and other structures under biaxial loading condition.

## 5. Conclusions

To enable the application of two-parameter EPFM approaches on sensor structure design as well as other engineering applications, an *T*-stress-based estimate method was developed by the authors under an SSY condition, which determines the solutions of constraint parameter *A* in the *J-A* two-parameter approach directly from structure *T*-stress, both conveniently and effectively. Based on the relation curve shape similarity between parameter *A* and *T*-stress, a simplified formation of the *T*-stress-based estimate was also proposed under a uniaxial external loading condition, which enabled much more convenient and quick estimates of constraint parameter *A* values than its original format. 

Sensor components and other engineering structures generally endure biaxial external loading instead of the uniaxial one. In the current work, through analysis and discussion of specimens SECP, CCP, and DECP, the simplified format of *T*-stress-based estimate method was extended to a biaxial loading condition for sensor structure design and other engineering applications.

Through an extensive comparison between the predicted values and the numerical solutions of constraint parameter *A* for SECP, CCP, and DECP, the extension of simplified format *T*-stress-based estimate method to biaxial loading condition was validated. Based on the biaxial loading ratio *λ* = 0.5 and 1.0, the validation comparison was implemented based on crack specimens SECP, CCP, and DECP, with the relative crack length *a/W* = 0.1, 0.3, 0.5, and 0.7 for various hardening exponents *n* = 3, 4, 5, 7, and 10, respectively, covering a wide variety of material and geometric properties.

## Figures and Tables

**Table 1:** Parameter *A* values from FEA results for MBL formulation.

*T*/*σ*_0_	*n* = 3	*n* = 4	*n* = 5	*n* = 7	*n* = 10
−0.8	1.2297	0.8371	0.6433	0.4857	0.4189
−0.6	1.1626	0.7772	0.5913	0.4365	0.3659
−0.4	1.0866	0.7074	0.5254	0.3757	0.3050
−0.2	0.9959	0.6264	0.4529	0.3114	0.2429
0.0	0.8984	0.5432	0.3803	0.2489	0.1838
0.2	0.8077	0.4639	0.3114	0.1906	0.1298
0.4	0.7330	0.3957	0.2516	0.1409	0.0867
0.6	0.6767	0.3405	0.2032	0.0998	0.0522
0.8	0.6257	0.3011	0.1701	0.0652	0.0213

**Table 2:** Values of coefficients for one-to-one *A-T* relationship polynomials.

	*n* = 3	*n* = 4	*n* = 5	*n* = 7	*n* = 10
*A_SSY_*	0.8984	0.5432	0.3803	0.2489	0.1838
*m*_1_	−0.4588	−0.4064	−0.3581	−0.3039	−0.2808
*m*_2_	0.0443	0.0398	0.0412	0.0415	0.0570
*m*_3_	0.1300	0.1124	0.0972	0.0643	0.0509

**Table 3:** Maximum applicable load ratio (*σ*/*σ*_0_) of simplified format of *T*-stress-based estimate under biaxial loading.

		*λ* = 0.5				*λ* = 1.0			
Model	*n*	*a/W* = 0.1	*a/W* = 0.3	*a/W* = 0.5	*a/W* = 0.7	*a/W* = 0.1	*a/W* = 0.3	*a/W* = 0.5	*a/W* = 0.7
SECP	3	2.100	1.400	0.380	0.095	0.600	1.050	0.450	0.100
	4	1.900	1.150	0.350	0.085	0.600	0.950	0.400	0.090
	5	1.800	0.950	0.320	0.080	0.600	0.950	0.380	0.080
	7	1.700	0.850	0.320	0.070	0.600	1.000	0.350	0.070
	10	1.600	0.810	0.320	0.060	0.500	0.950	0.300	0.060
CCP	3	2.200	1.800	0.450	0.250	1.200	1.300	1.450	0.400
	4	1.800	1.800	0.550	0.250	1.000	1.200	1.400	0.350
	5	1.500	1.750	0.650	0.250	1.000	1.000	1.300	0.400
	7	1.200	1.500	1.200	0.350	1.000	1.000	1.200	0.500
	10	1.000	1.200	1.150	0.400	1.000	1.000	1.100	0.500
DECP	3	2.100	2.100	1.250	0.700	0.650	0.650	0.750	1.050
	4	2.000	2.200	1.250	0.600	0.500	0.500	0.650	1.100
	5	2.000	2.200	1.050	0.550	0.650	0.650	0.650	1.200
	7	1.900	2.000	0.950	0.550	0.750	0.750	0.850	0.650
	10	1.800	1.700	0.940	0.540	0.750	0.900	1.050	0.550

**Table 4:** Maximum applicable load ratio (*σ*/*σ*_0_) of *T*-stress-based estimate under biaxial loading.

		*λ* = 0.5				*λ* = 1.0			
Model	*n*	*a/W* = 0.1	*a/W* = 0.3	*a/W* = 0.5	*a/W* = 0.7	*a/W* = 0.1	*a/W* = 0.3	*a/W* = 0.5	*a/W* = 0.7
SECP	3	1.800	1.400	0.350	0.080	1.000	1.400	0.350	0.080
	4	1.700	1.300	0.320	0.070	0.900	1.200	0.300	0.070
	5	1.600	1.100	0.320	0.070	0.850	1.150	0.300	0.070
	7	1.600	0.900	0.320	0.070	0.750	1.100	0.300	0.060
	10	1.600	0.810	0.320	0.060	0.500	0.950	0.300	0.060
CCP	3	1.800	1.800	1.200	0.640	1.200	1.300	1.300	0.750
	4	1.500	1.800	1.200	0.400	1.000	1.000	1.200	0.750
	5	1.200	1.750	1.200	0.400	1.000	1.000	1.200	0.750
	7	1.200	1.500	1.200	0.400	1.000	1.000	1.100	0.540
	10	1.000	1.200	1.150	0.400	1.000	1.000	1.100	0.500
DECP	3	2.000	2.000	1.250	0.700	1.000	1.100	1.200	1.200
	4	2.000	2.200	1.250	0.650	0.900	1.000	1.100	0.870
	5	1.900	2.200	1.150	0.550	0.900	0.900	1.050	0.650
	7	1.900	2.000	0.950	0.550	0.900	0.900	1.050	0.550
	10	1.800	1.700	0.940	0.540	0.750	0.900	1.050	0.550
